# Effectiveness of adalimumab in treating patients with ankylosing spondylitis associated with enthesitis and peripheral arthritis

**DOI:** 10.1186/ar2953

**Published:** 2010-03-15

**Authors:** Martin Rudwaleit, Pascal Claudepierre, Martina Kron, Sonja Kary, Robert Wong, Hartmut Kupper

**Affiliations:** 1 Charité-University Medicine Berlin, Benjamin Franklin Campus, Medical Department I, Rheumatology, Hindenburgdamm 30, 12203 Berlin, Germany; 2 Service de Rhumatologie, Hôpital Henri Mondor-APHP, Université Paris XII, avenue du Maréchal de Lattre de Tassigny 51, 94010 Créteil, France; 3 Abbott GmbH & Co KG, Knollstraße 50, 67061 Ludwigshafen, Germany; 4 University of Medicine and Dentistry of New Jersey, 189 New Street, New Brunswick, NJ 08901-1954, USA; 5 Formerly Abbott Laboratories, Parsippany, NJ, USA

## Abstract

**Introduction:**

The purpose of this study was to investigate the effectiveness of adalimumab in enthesitis and peripheral arthritis in patients with ankylosing spondylitis (AS).

**Methods:**

Adults with active AS (Bath ankylosing spondylitis disease activity index [BASDAI] ≥ 4) received adalimumab 40 mg every other week with standard antirheumatic therapies in a 12-week, open-label study. Effectiveness in enthesitis was assessed using the Maastricht ankylosing spondylitis enthesitis score (MASES, 0-13) and by examining the plantar fascia in patients with enthesitis (≥ 1 inflamed enthesis) at baseline; effectiveness in peripheral arthritis was evaluated using tender and swollen joint counts (TJC, 0-46; SJC, 0-44) in patients with peripheral arthritis (≥ 1 swollen joint) at baseline. Overall effectiveness measures included Assessment of SpondyloArthritis International Society 20% response (ASAS20).

**Results:**

Of 1,250 patients enrolled, 686 had enthesitis and 281 had peripheral arthritis. In 667 patients with MASES ≥ 1 at baseline, the median MASES was reduced from 5 at baseline to 1 at week 12. At week 12, inflammation of the plantar fascia ceased in 122 of 173 patients with inflammation at baseline. The median TJC in 281 patients with SJC ≥ 1 at baseline was reduced from 5 at baseline to 1 at week 12; the median SJC improved from 2 to 0. ASAS20 responses were achieved by 70.5% of 457 patients with no enthesitis and no arthritis; 71.0% of 512 patients with only enthesitis; 68.0% of 107 patients with only arthritis; and 66.7% of 174 patients with both.

**Conclusions:**

Treatment with adalimumab improved enthesitis and peripheral arthritis in patients with active AS.

**Trial registration:**

ClinicalTrials.gov NCT00478660.

## Introduction

In addition to chronic inflammation of the spine, extra-axial manifestations are common features in patients with ankylosing spondylitis (AS). Enthesitis and peripheral arthritis, predominantly of the lower limbs, occur in up to 50% of patients with AS during the course of the disease [1-5]. These extra-axial manifestations of AS contribute to the burden of the disease [6,7].

Nonsteroidal anti-inflammatory drugs (NSAIDs) remain first-line agents for the treatment of AS and can be used for the treatment of enthesitis [8]. Disease-modifying antirheumatic drugs (DMARDs) do not have a satisfactory effect on axial disease. Sulfasalazine has some effect on extra-axial arthritis [9] but its benefit for treating enthesitis does not outweigh its risks [8]. Tumor necrosis factor (TNF) antagonists, including the monoclonal antibodies adalimumab and infliximab and the TNF-receptor construct etanercept, are highly effective agents for the treatment of patients who have active AS despite NSAID treatment [9-18].

In a 24-week, randomized, double-blind, placebo-controlled study of patients with active AS, 152 adalimumab-treated patients experienced a significant reduction in enthesitis compared with 81 placebo-treated patients but no significant improvement in peripheral arthritis [15]. Similarly, other randomized controlled trials (RCTs) of TNF antagonists have not consistently demonstrated significant improvements in both enthesitis and peripheral arthritis for TNF-antagonist-treated patients compared with placebo-treated patients [10,12,14,16,17]. Only in one RCT of infliximab in AS were significant improvements in both tender joint count (TJC) and swollen joint count (SJC) observed [10]. We evaluated the effects of adalimumab on enthesitis and peripheral arthritis in a large cohort of 1,250 patients with active AS who were enrolled in the open-label RHAPSODY (Review of Safety and Effectiveness with Adalimumab in Patients with Active Ankylosing Spondylitis) study [1].

## Materials and methods

The patient sample and methods of the RHAPSODY study, an open-label, multicenter study conducted at 211 centers in 15 European countries, were previously described in detail [1]. Independent ethics committees at all participating centers approved the study, and all participating patients gave written informed consent.

### Patients

Patients who were at least 18 years old and who had AS according to the modified 1984 New York Criteria for AS [19] and active disease defined by a Bath Ankylosing Spondylitis Disease Activity Index (BASDAI) score of at least 4 [20] were eligible for this study if at least one NSAID or (if stipulated by national guidelines) at least two NSAIDs had failed to control their disease. Patients were permitted to continue current AS therapy with NSAIDs, DMARDs, analgesics, and glucocorticoids (≤ 10 mg/d of prednisone equivalent) during the study. Intra-articular injections or infiltrations of extra-axial joints and tendons were not permitted within 28 days and injections of sacroiliac joints were not permitted within 14 days before screening or during the study. Previous anti-TNF therapy was permitted provided that etanercept had been discontinued at least 3 weeks or infliximab had been discontinued at least 2 months before the first adalimumab injection.

### Study design

At baseline, clinicians documented the presence or absence of enthesitis and peripheral arthritis. Enthesitis was defined as at least 1 inflamed enthesis in the Maastricht Ankylosing Spondylitis Enthesitis Score (MASES) (0 to 13) [21] or of the plantar fascia of the foot. Peripheral arthritis was defined as an SJC of at least 1 (0 to 44, excluding hip joints).

Patients self-administered 40 mg of adalimumab (Abbott Laboratories, Abbott Park, IL, USA) subcutaneously every other week for 12 weeks. The treatment effect of adalimumab for enthesitis was assessed by measuring the change from baseline to week 12 in the MASES (for patients with MASES of at least 1 at baseline) and the change from baseline to week 12 in the percentage of patients with enthesitis of the plantar fascia (for patients with inflammation of the plantar fascia at baseline). The treatment effect of adalimumab for peripheral arthritis was assessed by measuring the change in TJCs (0 to 46) and SJCs from baseline to weeks 2, 6, and 12. Overall adalimumab effectiveness was measured using the Assessment of SpondyloArthritis International Society 20% and 40% responses (ASAS20 and ASAS40, respectively) [20,22] and using a 50% improvement in the BASDAI (BASDAI 50) [23,24]. In addition, absolute values at each study visit (weeks 2, 6, and 12, as applicable) and changes from baseline were calculated for BASDAI (0 to 10) [20], Bath Ankylosing Spondylitis Functional Index (BASFI) (0 to 10) [25], the Patient's Global Assessment of disease activity (PaGA) (based on a visual analog scale of 0 to 100 mm), and C-reactive protein (CRP) serum concentration.

### Statistical analysis

Patients who had received at least one injection of adalimumab were included in the analyses. Observed data were used for all effectiveness analyses, and week 12 was specified as the endpoint. Patients with MASES of at least 1 and patients with SJC of at least 1 were stratified by sex (male or female), HLA-B27 positivity (positive or negative), and concomitant DMARD therapy (yes or no) to analyze changes in MASES, TJC, and SJC. The effects of adalimumab on AS disease activity and functional disability were evaluated in four subgroups: (a) patients with no enthesitis and no peripheral arthritis, (b) patients with enthesitis and no peripheral arthritis, (c) patients with peripheral arthritis and no enthesitis, and (d) patients with both enthesitis and peripheral arthritis.

Descriptive analyses were performed by calculating counts and percentages for qualitative data and by calculating medians and first and third quartiles for quantitative data. For the three patient groups without arthritis and enthesitis, with either peripheral arthritis or enthesitis, and with both, the Jonckheere-Terpstra test was applied to test for trends in baseline values and absolute changes from baseline to week 12 in BASDAI, BASFI, PaGA, and CRP. For the median absolute change from baseline to week 12 in MASES, TJC, SJC, BASDAI, BASFI, PaGA, and CRP, nonparametric 95% confidence intervals (CIs) were calculated. Correlations between improvements in AS overall (measured by changes in BASDAI and in BASFI) and improvements in extra-axial manifestations (measured by changes in MASES, plantar fascia, TJC, and SJC) at week 12 were assessed using the Spearman rank order correlation coefficient.

## Results

### Patient disposition

**Figure 1:**
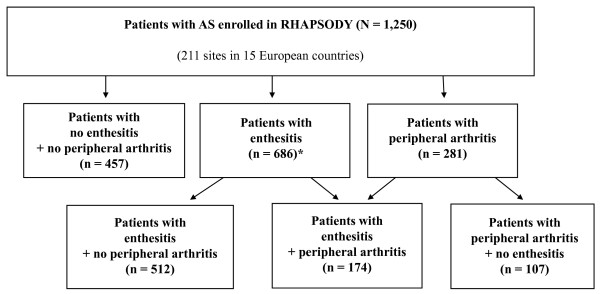
**Distribution of enthesitis or peripheral arthritis or both in patients with ankylosing spondylitis (AS) (n = 1,250)**. Enthesitis is defined as at least 1 inflamed enthesis in Maastricht Ankylosing Spondylitis Enthesitis Score (MASES) or plantar fascia, and peripheral arthritis is defined as at least 1 swollen joint in a 44-joint count. Data are observed values. *Six hundred sixty-seven patients with enthesitis in MASES and 173 patients with enthesitis of fascia plantaris (19 patients had enthesitis of fascia plantaris without enthesitis in MASES). RHAPSODY, Review of Safety and Effectiveness with Adalimumab in Patients with Active Ankylosing Spondylitis.

**Figure 2:**
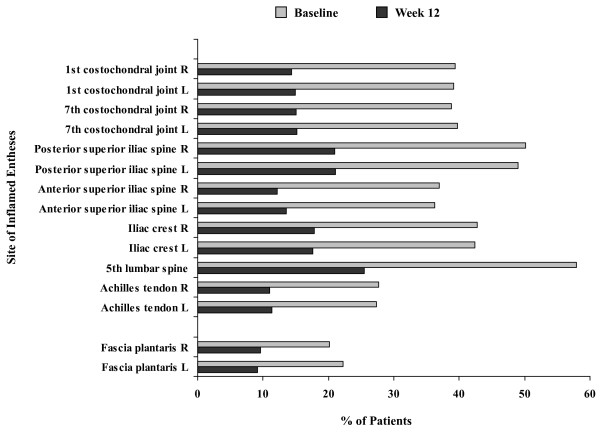
**Percentages of patients with inflamed entheses at baseline and week 12 by enthesitis site (n = 686)**. Enthesitis is defined as at least 1 inflamed enthesis in Maastricht Ankylosing Spondylitis Enthesitis Score or plantar fascia and includes patients with ankylosing spondylitis and enthesitis. Data are observed values.

A total of 1,250 patients with AS were enrolled. Through week 12, 7.3% of the patients discontinued adalimumab therapy [1]. Of enrolled patients, 457 (36.6%) patients had no enthesitis and no peripheral arthritis at baseline (Figure 1). Enthesitis (MASES or plantar fascia or both of at least 1) was reported in 686 (54.9%) patients. At least one inflamed enthesis in MASES was documented for 667 patients, and inflammation of the plantar fascia was reported for 173 patients, including 19 patients without enthesitis in MASES. The distribution of enthesitis sites at baseline is shown in Figure 2. Of the 281 (22.5%) patients with peripheral arthritis (SJC of at least 1) at baseline, 96 had tenderness of the hip joint, indicating possible hip involvement. Enthesitis without peripheral arthritis was documented in 512 (41.0%) patients; arthritis without enthesitis was reported in 107 (8.6%) patients. Both enthesitis and peripheral arthritis were present in 174 of 1,250 (13.9%) patients.

### Baseline characteristics

#### Patients with enthesitis or peripheral arthritis or both

The mean age of patients and the mean duration of AS were similar between patient subgroups, irrespective of the presence of enthesitis and arthritis (Table 1). The percentage of men was lower in the patient subgroups with enthesitis or peripheral arthritis or both (range, 58.0% to 69.2%) than in the subgroup with no enthesitis and no arthritis (80.7%). The percentage of patients with HLA-B27 was lower in the subgroup with both enthesitis and peripheral arthritis (76.0%) than in the other subgroups (range, 82.2% to 84.1%). The patient subgroup with peripheral arthritis and no enthesitis had the greatest percentages of patients receiving concomitant therapy with DMARDs (45.8%) or glucocorticoids (29.9%). Median BASDAI, BASFI, and PaGA scores were lower in patients with no enthesitis and no arthritis than in patients with enthesitis or peripheral arthritis or both (*P *< 0.005) (Table 2).

**Table 1:** Baseline characteristics in patients with ankylosing spondylitis by subgroup of enthesitis or peripheral arthritis or both

	Patient subgroups			
	
Characteristic	No enthesitis + no arthritis (n = 457)	Enthesitis + no arthritis (n = 512)	Arthritis + no enthesitis (n = 107)	Enthesitis + arthritis (n = 174)
Age, years	43.1 ± 11.2	42.7 ± 11.1	46.0 ± 11.9	46.1 ± 12.3
Males, percentage	80.7	67.8	69.2	58.0
HLA-B27-positive, percentage	83.7	82.2	84.1	76.0
AS duration, years	10.9 ± 9.4	10.5 ± 9.7	12.3 ± 10.6	11.8 ± 10.5
Advanced AS, percentage	25.2	28.4	27.6	26.5
Concomitant NSAIDs, percentage	74.0	76.8	73.8	68.4
Concomitant DMARDs, percentage	21.7	22.1	45.8	35.6
Concomitant glucocorticoids^a^, percentage	10.1	10.9	29.9	20.1
Prior TNF-antagonist therapy, percentage	25.8	25.0	27.1	29.3

Data are mean ± standard deviation unless otherwise noted. ^a^Systemic treatment with not more than 10 mg of prednisone equivalent daily. AS, ankylosing spondylitis; DMARD, disease-modifying antirheumatic drug; NSAID, nonsteroidal anti-inflammatory drug; TNF, tumor necrosis factor.

**Table 2:** Effectiveness of adalimumab at week 12 in patients with ankylosing spondylitis by subgroup of enthesitis or peripheral arthritis or both

	No enthesitis + no arthritis (n = 457)	Enthesitis + no arthritis (n = 512)	Arthritis + no enthesitis (n = 107)	Enthesitis + arthritis (n = 174)	*P *value^a^
		
	Median First quartile, third quartile [95% confidence interval]				
BASDAI, 0 to 10					

Baseline	5.9 4.9, 6.9	6.4 5.4, 7.5	6.7 5.7, 7.7	6.6 5.7, 7.6	< 0.001
Week 12	2.1 0.8, 4.1	2.6 1.1, 5.1	3.1 1.5, 4.8	2.7 1.2, 5.0	
Change from baseline to week12	-3.6 -4.8, -1.7 [-3.8 to -3.3]	-3.4 -4.9, -1.5 [-3.7 to -3.1]	-3.1 -5.0, -1.8 [-4.0 to -2.7]	-3.5 -4.9, -1.7 [-3.7 to -2.9]	0.813

BASFI, 0 to 10					

Baseline	4.9 3.4, 6.7	5.7 4.0, 7.3	5.8 3.7, 7.0	6.0 4.3, 7.5	< 0.001
Week 12	2.2 1.0, 4.3	2.7 1.0, 5.1	3.1 1.4, 5.3	2.8 1.3, 5.3	
Change from baseline to week12	-1.9 -3.6, -0.7 [-2.1 to -1.7]	-2.1 -3.8, -0.6 [-2.4 to -1.7]	-1.8 -3.2, -0.7 [-2.2 to -1.5]	-1.9 -3.7, -0.5 [-2.2 to -1.5]	0.828

Patient's Global Assessment of disease activity, 0- to 100-mm visual analog scale					

Baseline	67 49, 80	71 53, 83	72 54, 81	72 53, 83	0.004
Week 12	21 8, 46	24 8, 52	25 11, 48	26 10, 52	
Change from baseline to week12	-35 -58, -14 [-40 to -32]	-38 -59, -15 [-42 to -34]	-39 -61, -13 [-48 to -26]	-33 -57, -12 [-41 to -24]	0.806

C-reactive protein^b^, mg/dL					

Baseline	1.2 0.6, 2.5	1.2 0.5, 2.4	1.5 0.8,3.6	1.3 0.6, 3.4	0.448
Week 12	0.4 0.2, 0.8	0.4 0.2, 0.8	0.6 0.2, 1.5	0.4 0.1, 0.9	
Change from baseline to week12	-0.7 -2.1, -0.1 [-0.9 to -0.6]	-0.8 -1.7, -0.2 [-1.0 to -0.7]	-1.4 -1.7, -0.1 [-1.0 to -0.4]	-0.7 -2.6, -0.1 [-1.3 to -0.4]	0.857

^a^*P *values (two-sided) are based on Jonckheere-Terpstra tests for trends between subgroups without enthesitis and peripheral arthritis, with either enthesitis or peripheral arthritis, and with both. ^b^Reference value is 0.4 mg/dL. BASDAI, Bath Ankylosing Spondylitis Disease Activity Index; BASFI, Bath Ankylosing Spondylitis Functional Index.

#### Patients with MASES of at least 1 at baseline

The median MASES was 5 for the 667 patients with MASES of at least 1 at baseline (Table 3). The median MASESs were 4 for 432 male patients and 7 for 236 female patients, 5 for both 523 HLA-B27-positive patients and 126 HLA-B27-negative patients, and 5 for 499 patients without concomitant DMARD treatment at baseline and 4 for 168 patients with concomitant DMARD therapy at baseline.

**Table 3:** MASES, tender joint count, and swollen joint count by sex, HLA-B27 positivity, and concomitant DMARD use

	All	Male	Female	HLA-B27-negative	HLA-B27-positive	No concomitant DMARD at baseline	Concomitant DMARD at baseline
	
	Median First quartile, third quartile [95% confidence interval]						
Patients with enthesitis, MASES ≥ 1 at baseline							
MASES: 0 to 13, number (percentage)	667 (100)	431 (64.6)	236 (35.4)	126 (18.9)^a^	523 (78.4)^a^	499 (74.8)	168 (25.2)
Baseline	5 2, 8	4 2, 7	7 4, 9	5 2, 9	5 2, 8	5 2, 8	4 2, 8
Week 12	1 0, 4	0 0, 2	1 0, 5	1 0, 5	0 0, 3	0 0, 3	1 0, 4
Change from baseline to week 12	-2 -5,-1 [-3 to -2]	-2 -5,-1 [-3 to -2]	-3 -6,-1 [-4 to -2]	-2 -5,-1 [-3 to -2]	-3 -6,-1 [-3 to -2]	-3 -5,-1 [-3 to -2]	-2 -5,-1 [-5 to -2]

Patients with peripheral arthritis, SJC ≥ 1 at baseline							
TJC: 0 to 46, number (percentage)	281 (100)	175 (62.2)	106 (37.7)	57 (20.3)^a^	217 (77.2)^a^	170 (60.5)	111 (39.5)
Baseline	5 2, 12	4 2, 10	7 3, 14	6 3, 13	5 2, 12	4 2, 10	6 3, 13
Week 12	1 0, 3	1 0, 3	1 0, 4	1 0, 4	0 0, 3	0 0, 3	1 0, 4
Change from baseline to week 12	-3 -8,-1 [-4 to -2]	-2 -6,-1 [-3 to -2]	-4 -10,-2 [-6 to -3]	-3 -10,-1 [-6 to -2]	-3 -7,-1 [-4 to -2]	-2 -7,-1 [-3 to -2]	-3 -9,-2 [-4 to -2]

SJC: 0 to 44, number (percentage)	281 (100)	175 (62.2)	106 (37.7)	57 (20.3)^a^	217 (77.2)^a^	170 (60.5)	111 (39.5)
Baseline	2 1, 4	2 1, 3	2 1, 6	2 1, 6	2 1, 4	2 1, 3	2 1, 6
Week 12	0 0, 1	0 0, 1	0 0, 1	0 0, 1	0 0, 1	0 0, 1	0 0, 1
Change from baseline to week 12	-1 -3,-1 [-2 to -1]	-1 -3,-1 [-2 to -1]	-2 -4,-1 [-2 to -1]	-1 -4,-1 [-2 to -1]	-1 -3,-1 [-2 to -1]	-1 -2,-1 [-1 to -1]	-2 -4,-1 [-2 to -2]

^a^Because of missing data, the percentages of patients with HLA-B27 status do not sum to 100%. DMARD, disease-modifying antirheumatic drug; MASES, Maastricht Ankylosing Spondylitis Enthesitis Score; SJC, swollen joint count; TJC, tender joint count.

#### Patients with enthesitis of plantar fascia

The percentages of patients with enthesitis of the plantar fascia were 11.8% in men (105 of 891 men) and 18.9% in women (68 of 359 women).

#### Patients with a swollen joint count of at least 1 at baseline

The median TJCs and SJCs were 5 and 2, respectively, at baseline for 281 patients with an SJC of at least 1 (Table 3). Knees (35% of joints), ankles (35%), and hips (30%) were most frequently affected by tenderness, whereas of the joints of the upper limbs, the greatest percentages of joint tenderness were observed in the wrists (28%) and shoulders (27%). Similarly, of the joints of the lower limbs, the greatest percentages of joint swelling were reported for knees (25%) and ankles (22%) compared with 19% for the wrists. (The hips were not evaluated for swelling.) For fingers and toes, swelling was more frequently reported for the metacarpophalangeal joints and proximal interphalangeal joints (4% to 17%) than for the metatarsophalangeal (MTP) joints (1% to 6%), probably because swelling in the MTP joints is difficult to evaluate. The median TJCs were 4 for 175 male patients and 7 for 106 female patients, 5 for 217 HLA-B27-positive patients and 6 for 57 HLA-B27-negative patients, and 4 for 170 patients not receiving concomitant DMARD therapy and 6 for 111 receiving concomitant DMARD therapy. The median SJC was 2, regardless of sex, HLA-B27, and DMARD treatment.

### Adalimumab effectiveness at week 12

#### Enthesitis

For patients with MASES of at least 1 at baseline, the median MASES decreased from 5 at baseline to 1 at week 12; the median change in MASES from baseline to week 12 was -2 (Table 3). The median MASESs at week 12 were 1 for women (median reduction, -3) and 0 for men (median reduction, -2). The improvement in MASES was independent of HLA-B27 positivity and concomitant DMARD therapy. Enthesitis of the plantar fascia resolved in 122 of 173 (70.5%) patients (77 men and 45 women) at week 12. Overall, similar improvements in enthesitis were observed across enthesitis sites (Figure 2).

#### Peripheral arthritis

After 12 weeks of treatment with adalimumab, the TJC for 281 patients with peripheral arthritis (SJC of at least 1) decreased from a median of 5 at baseline to 1, with a median reduction of -3 (Table 3). The SJC improved from a median of 2 at baseline to 0 at week 12, with a median reduction of -1. Rapid improvement in peripheral arthritis was observed, with reductions in the median TJC and SJC documented after a single injection of adalimumab; at week 2, the median TJC was 2 and the median SJC was 0.

#### Overall

**Figure 3:**
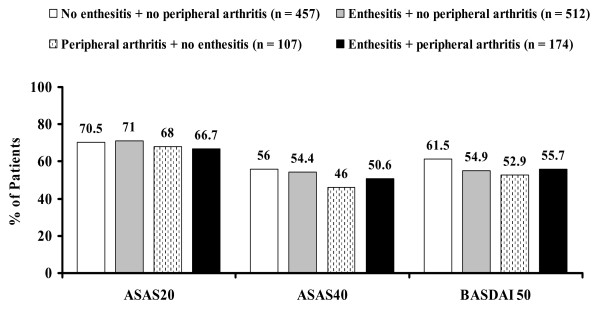
**Percentages of patients with ankylosing spondylitis achieving response at week 12**. Enthesitis is defined as at least 1 inflamed enthesis in Maastricht Ankylosing Spondylitis Enthesitis Score or plantar fascia, and peripheral arthritis is defined as at least 1 swollen joint in a 44-joint count. Data are observed values. ASAS20/40, Assessment of SpondyloArthritis international Society 20%/40% response; BASDAI 50, 50% improvement in the Bath Ankylosing Spondylitis Disease Activity Index.

As shown in Figure 3, the percentage of patients achieving ASAS20 response at week 12 was lowest in the subgroup of patients with both enthesitis and peripheral arthritis (66.7%) at baseline compared with the other patient subgroups (68.0 to 71.0%), whereas the percentage achieving ASAS40 response was lower in the subgroup with peripheral arthritis and no enthesitis (46.0%) compared with the others (50.6% to 56%). Similarly, the percentage achieving a BASDAI50 response was lower in patients with peripheral arthritis and no enthesitis (52.9%) than in other subgroups (54.9% to 61.5%). Median changes from baseline to week 12 and 95% CIs in BASDAI, BASFI, PaGA, and CRP were similar between patient subgroups (*P *> 0.8) (Table 2).

### Correlation between improvements in axial disease and improvements in enthesitis or peripheral arthritis or both

Improvement in AS disease activity and functional status, measured by BASDAI and BASFI, respectively, did not correlate with improvement in enthesitis, measured by MASES and inflammation of the plantar fascia, or improvement in peripheral arthritis, measured by TJC and by SJC. Spearman rank order correlation coefficients ranged from 0.03 to 0.24 across evaluations of the various combinations of measures.

## Discussion

Adalimumab effectiveness and safety were investigated in a large cohort of patients with AS in an open-label, uncontrolled, multinational study designed to reflect routine rheumatology practice [1]. The patients had long-standing, active AS with a broad range of disease manifestations typically associated with AS and were considered eligible for TNF-antagonist therapy [9]. Herein, we specifically assessed the effectiveness of adalimumab in AS patients with or without enthesitis or peripheral arthritis. The baseline frequency of current enthesitis (54.9%) in this population and that reported in a previous adalimumab RCT (74%) [15] are both markedly greater than the frequency reported in recent national observational studies (15% to 30%) [1-3], whereas the rate of current peripheral arthritis found in this study was in accordance with rates reported in the literature [2].

In this study, adalimumab therapy was associated with substantial improvement in enthesitis from baseline to week 12, as indicated by changes in MASES, and in inflammation across all examined entheses, including the plantar fascia. Our results are consistent with results of a previous RCT of adalimumab in 315 patients with AS [15] and with an RCT of infliximab [14], although comparison with the latter trial is limited by the use of disparate enthesitis scores.

In the present study, peripheral arthritis in the 281 patients who had at least 1 swollen joint at baseline improved rapidly and substantially during adalimumab therapy, with half of the patients reporting no swollen joints at week 12 (week-12 median TJC = 1 and SJC = 0). Because of the skewed distribution of values, median data are presented here. In a previous adalimumab RCT in which mean data were published [15], the mean change at week 12 in TJC was -0.8 and the mean change in SJC was -0.4. By comparison, in our study, the mean changes from baseline to week 12 were -4.9 for TJC and -2.9 for SJC, and the median changes were lower than these mean changes (TJC, -3 and SJC, -1). Significant decreases in TJC and SJC from baseline to week 12 have been reported for infliximab therapy for AS [10].

Women with AS had a greater number of inflamed joints and inflamed entheses at baseline than men. Although the presence of coexisting fibromyalgia in some of the female patients cannot be excluded, the pattern of enthesitis localization as measured by MASES and examination of the plantar fascia is typical for enthesitis in AS (Figure 2) [21,26]. Moreover, the finding that female patients improved as much as male patients after 12 weeks of adalimumab therapy suggests that coexisting fibromyalgia did not contribute substantially to the greater enthesitis count at baseline in women. The frequency of and improvement in enthesitis and peripheral arthritis appeared to be unaffected by HLA-B27 status and concomitant DMARD therapy.

The burden of disease at baseline, as measured by BASDAI, BASFI, and PaGA, was lower for patients with no enthesitis and no peripheral arthritis than for patients with either one or both of these manifestations (*P *< 0.005). These results are in accordance with previous literature reports [7,27,28]. Of note, two studies showed that a greater BASDAI in patients with peripheral arthritis was not associated with the joint-specific BASDAI items [27,29]. Although disease activity and physical impairment differed notably at baseline, the magnitude of improvement with adalimumab therapy in patients with enthesitis or peripheral arthritis or both was similar to that observed for patients without these extra-axial manifestations. At week 12, the median changes in BASDAI, BASFI, PaGA, and CRP were similar between all of the various subgroups, and no important differences in the degree of improvement were detected. However, slightly greater ASAS40 and BASDAI 50 response rates were observed for patients with no enthesitis and no arthritis than for those with enthesitis or arthritis or both. The results of this study of patients with AS categorized by enthesitis and arthritis are consistent with previous predictor analyses, which found that enthesitis and arthritis did not influence the effect of TNF-antagonist therapy [1].

We did not detect any correlation between improvement in BASDAI and BASFI with improvement in enthesitis or peripheral arthritis. In comparison with joint counts and enthesitis counts, which capture a single domain of AS that may entirely resolve, BASDAI and BASFI capture many disease domains of AS, each of which may respond differently to therapy.

## Conclusions

In this large cohort of patients with active AS, adalimumab treatment effectively reduced enthesitis and peripheral arthritis. Although the burden of disease is greater when AS is associated with enthesitis or peripheral arthritis or both, patients with these extra-axial manifestations benefited from adalimumab treatment as much as patients with axial disease only.

## Abbreviations

AS: ankylosing spondylitis; ASAS20/40: Assessment of SpondyloArthritis international Society 20%/40% response; BASDAI: Bath Ankylosing Spondylitis Disease Activity Index; BASDAI 50: 50% improvement in the Bath Ankylosing Spondylitis Disease Activity Index; BASFI: Bath Ankylosing Spondylitis Functional Index; CI: confidence interval; CRP: C-reactive protein; DMARD: disease-modifying antirheumatic drug; MASES: Maastricht Ankylosing Spondylitis Enthesitis Score; MTP: metatarsophalangeal; NSAID: nonsteroidal anti-inflammatory drug; PaGA: Patient's Global Assessment of disease activity; RCT: randomized controlled trial; RHAPSODY: Review of Safety and Effectiveness with Adalimumab in Patients with Active Ankylosing Spondylitis; SJC: swollen joint count; TJC: tender joint count; TNF: tumor necrosis factor.

## Competing interests

MR and PC were RHAPSODY study investigators. MR has received consulting fees, speaking fees, and honoraria from Abbott, MSD (München, Germany), Schering-Plough Corporation (Kenilworth, NJ, USA), Pfizer Inc (New York, NY, USA), and Wyeth (Madison, NJ, USA). MK and HK are employees of Abbott GmbH & Co KG (Ludwigshafen, Germany), an affiliate of Abbott Laboratories, and hold shares of Abbott stock. SK is a contractor of Abbott GmbH & Co KG. RW was an employee of Abbott Laboratories at the time this study and these analyses were completed and holds shares of Abbott stock.

## Authors' contributions

All authors contributed to manuscript development and reviewed and approved the content of the submitted manuscript.
